# Retraction: Capsaicin and Cold exposure promote EMT-mediated premetastatic niche formation to facilitate colorectal cancer metastasis

**DOI:** 10.7150/jca.96595

**Published:** 2024-04-09

**Authors:** Feifei Nong, Shangping Xing

**Affiliations:** 1 Department of Scientific Research, The First Affiliated Hospital of Guangxi University of Chinese Medicine, Guangxi University of Chinese Medicine, Nanning, 530024, China.; 2 Guangxi Key Laboratory of Molecular Biology of Preventive Medicine of Traditional Chinese Medicine, Nanning, 530024, China.; 3 Guangxi Key Laboratory of Bioactive Molecules Research and Evaluation, School of Pharmacy, Guangxi Medical University, Nanning, 530022, China.

Due to the fact that one another author was not included in the attribution of the published paper "Capsaicin and Cold exposure promote EMT-mediated premetastatic niche formation to facilitate colorectal cancer metastasis”, there is a conflict of interest among the authors. Therefore, we would like to retract the published paper and express our sincere apologies for the distress caused to the authors and readers concerned.

